# The prevalence of *Mycoplasma genitalium* (MG) and *Trichomonas vaginalis* (TV) at testing centers in Belgium, Germany, Spain, and the UK using the cobas TV/MG molecular assay

**DOI:** 10.1007/s10096-022-04521-5

**Published:** 2022-11-11

**Authors:** Michael D. Perry, Sophie Jones, Alexander Bertram, Adolfo de Salazar, Antonio Barrientos-Durán, Gilberte Schiettekatte, Michael Lewinski, Rodney Arcenas, Avneet Hansra, Merlin Njoya, Federico García

**Affiliations:** 1 Public Health Wales Microbiology, University Hospital of Wales, Heath Park, Cardiff, CF14 4XW UK; 2 Amedes MVZ Wagnerstibbe für Laboratoriumsmedizin, Hämostaseologie, Humangenetik und Mikrobiologie, Hannover, Germany; 3 Department of Clinical Microbiology, Hospital Universitario Clínico San Cecilio, Instituto de Investigación Ibs, Granada, Spain; 4 Centrum voor Medische Analyse, Herentals, Belgium; 5 Roche Molecular Systems, Inc., Pleasanton, CA USA; 6 CIBER de Enfermedades Infecciosas (CIBERINFEC), ISCIII, Madrid, Spain

**Keywords:** cobas TV/MG, *Mycoplasma genitalium*, *Trichomonas vaginalis*, Molecular diagnostics, PCR, Public health

## Abstract

*Mycoplasma genitalium* (MG) and *Trichomonas vaginalis* (TV) can lead to long-term sequelae in males and females; however, global prevalence data vary between geographical regions, as these sexually transmitted infections are not included in routine screening. The objective of this study was to use the cobas^®^ TV/MG assay to assess the point prevalence of TV and MG in specimens from men and women over a broad European geographical area. Urine, vaginal, endocervical, and rectal samples were collected from patients aged ≥ 18 years receiving *Chlamydia trachomatis* (CT) and/or *Neisseria gonorrhoeae* (NG) screening as per local standard of care at sites in Belgium, Germany, Spain, and the UK (Wales). Remnant samples were assessed using the cobas TV/MG assay. Analysis of 2795 samples showed that MG prevalence varied slightly across female sample types (range: 1.7–5.8%; *p* = 0.0042). MG prevalence was higher in male rectal samples (12.5%) than in male urine samples (3.9%; *p* < 0.0001). TV prevalence was low in male (0.8%; 12/1535) and female (1.3%; 16/1260) samples across all sites. Co-infection of TV/MG with CT or NG was 10.0% (19/190) and 9.6% (7/73), respectively, in both male and female samples. MG and TV prevalence rates were comparable to the published literature in Europe. MG prevalence was highest in male rectal samples; as rectal testing is an off-label use of the cobas TV/MG assay, the clinical utility of this assay for rectal testing should be further investigated.

**Supplementary Information:**

The online version contains supplementary material available at 10.1007/s10096-022-04521-5.

## Introduction

*Mycoplasma genitalium* (MG) and *Trichomonas vaginalis* (TV) are common sexually transmitted infections (STIs) that can affect the urogenital tract, leading to long-term conditions such as cervicitis in women and urethritis in men [1, 2]. Additionally, MG is associated with pelvic inflammatory disease (PID) [2], and TV is associated with an increased risk of PID in women [1]. These infections have also been linked to increased risk of human immunodeficiency virus (HIV) acquisition and transmission [3, 4].

The prevalence of MG and TV infection varies depending on geographical region, and the presence of risk factors such as ethnicity, younger age, HIV seropositivity, smoking, and high-risk sexual behavior [1, 5–7]. Globally, the prevalence of MG is estimated to range from 9 to > 50% in high-risk groups [5], and the infection is detected in approximately 1 to 3.3% of men and women in the general population [2]. In patients attending STI clinics, the prevalence estimates for MG range from 1.9 to 36.5% according to geographic location [8–12]. Macrolide-resistant MG infections are a major concern, with high rates of macrolide antibiotic resistance being detected globally [2, 5, 13, 14], including in specimens from asymptomatic MG-infected participants [15]. This highlights the challenge of antimicrobial resistance for MG management and control, the need for reassessment of current diagnostic and treatment methods [5], and development of further optimized strategies in the future. In 2020, the WHO estimated that there were 156 million new TV infections [16]. Prevalence estimates from STI clinics range widely from 0.6 to 26% [1, 10, 11, 17], with some of the highest rates seen in African countries [1]. However, as most routine screening programs for STIs do not currently include TV and MG [11], the prevalence of these infections is likely to be underestimated.

Transmission of TV and MG is by mucosal contact, most commonly through sexual intercourse, and can be detected in urine samples (first void) or genital swabs [2, 18]. Rectal samples are also useful for the detection of MG in men who have sex with men (MSM) [14], in whom up to 70% of MG infections may be missed if this site is not sampled, and rectal MG infection in women at high risk is not uncommon [2]. Whilst there are currently limited data regarding TV colonization of the rectum, some studies have detected TV in rectal samples, including from MSM [19] and women [20].

Detection of TV infection has historically relied on microscopy and culture, but microscopy, despite being cost-effective, has low sensitivity, and culture is time-consuming and complex [21]. Before the development of nucleic acid amplification tests (NAATs), there was no routinely available and reliable test for diagnosing MG infection, due to difficulties in culturing this fastidious organism [7, 22]. The use of NAATs has enabled more sensitive and specific detection of these organisms [22] and provides a realistic option for screening, if deemed necessary, in the future. The cobas^®^ TV/MG assay for use on the cobas 6800/8800 systems is an automated, qualitative in vitro diagnostic test, that utilizes real-time polymerase chain reaction (PCR), for the direct detection of TV and/or MG DNA [23].

The objectives of this study were to assess the point prevalence of both MG and TV over a broad European geographical area using the cobas TV/MG assay in urogenital and rectal specimens from both men and women, as well as determine co-infections of MG or TV with *Chlamydia trachomatis* (CT), *Neisseria gonorrhoeae* (NG), HIV, or syphilis.

## Materials and methods

### Patient population and ethics

Samples were included from patients aged 18 years and over who were suitable for CT or NG screening according to local guidelines. The study was conducted in compliance with International Conference on Harmonisation (ICH) guidelines and Good Clinical Practice, and the protocol was approved by the Institutional Review Board (IRB) codes: 1559-N-18, UK NHS Health Research Authority Research Ethics Committee Reference Number – 18/EE/0334, 0175-N-19, CEIM/CEI Provincial de Granada, Comunidad Autónoma de Andalucía, Spain, EC UZA 18/47/545.

### Specimen collection

Remnant de-identified samples collected during standard-practice CT/NG testing were used. First-pass urine collection was used for urogenital testing in males. Depending on the standard of care for testing females at individual centers, first-pass urine, endocervical swabs, or vaginal swabs were collected. Anorectal swabs collected from both males and females were also analyzed. Sample collection was performed from July 2018 to April 2019. Specimens were collected at four sites: Amedes, Germany (AME); Centrum voor Medische Analyse, Belgium (CMA); Public Health Wales, Cardiff, UK (PHW); and Hospital Universitario Clínico San Cecilio, Spain (USC). Specimens were stabilized in cobas PCR media for testing.

If urine specimens could not be transferred to a cobas urine collection tube immediately after collection, they were stored at 2–30 °C for up to 24 h. Each specimen was required to be of sufficient volume, for testing up to five times, to be included in the study.

### Sample testing

Specimens were tested with the cobas TV/MG assay for use on the cobas 6800/8800 Systems (Roche Molecular Systems, Branchburg, NJ, USA), following the manufacturer’s recommended Instructions for Use (IFU). The performance of the cobas TV/MG assay for use on the cobas 6800/8800 Systems has been described previously and can reliably detect TV [24] and MG [25]. Sample testing was performed from December 2018 to April 2019.

### Data analysis

For each sample, where ethics review boards permitted and where information was available, the following data were collected: age, sex, ethnicity, specimen type, CT/NG result, HIV status, syphilis status, MSM, whether the patient was symptomatic, and the collection facility type. If clinical data were not available, the results were listed as unknown. Due to the remnant nature of the samples, symptom status was unfortunately unavailable in the majority of cases. Where available, it was not well characterized since there was no uniform symptomatic/asymptomatic definition due to different data collection criteria for the individual centers.

Prevalence and 95% score confidence intervals (CIs) were calculated from the number of positive samples as a proportion of the number of samples assessed for sample type, testing location, collection clinic type, and co-infections (if known). Prevalence was compared between specimen types within each sex, using the Fisher exact test, and between sex across common specimen types using the Cochran Mantel–Haenszel test. A sample size of 300 subjects ensured a level of precision (half width of the 95% score CIs) ranging from ± 1.7 to ± 3.4% for prevalence rates ranging from 2 to 10%, respectively. All data analyses were performed using SAS/STAT^®^ software.

## Results

### Sample characteristics

Of the 2874 samples that were evaluated, 2798 (97.4%) were eligible and 76 (2.6%) were excluded as they were from subjects aged under 18 years. From the 2798 eligible samples, the cobas TV/MG assay was not able to generate a valid result for 3 samples (0.11%). Therefore, there were 2795 samples with evaluable results, of which 54.9% were from men and 45.1% were from women (Table 1). Most samples were collected at STI clinics (45.6%) and in primary care (29.4%). Among samples for which symptom/infection status was obtained, most were asymptomatic (*n* = 752/1079; 69.7%), HIV negative (*n* = 1477/1571; 94.0%), and syphilis negative (*n* = 1322/1482; 89.2%); although these data were unknown or unobtainable for 61.4%, 43.8%, and 47.0% of samples, respectively, as they were not collected as part of the routine assessment at some locations.

**Table 1:** Demographics of evaluable samples

Characteristic	Samples
Total, *n*	2795
Age of subject, years	
Mean ± SD Median (range)	32.1 ± 13.2 28 (18–91)
Sex, (%)	
Female samples Male samples	1260 (45.1) 1535 (54.9)
Symptom status, *n* (%)	
Symptomatic Asymptomatic Unknown	327 (11.7) 752 (26.9) 1716 (61.4)
HIV status, *n* (%)	
HIV positive HIV negative Unknown	94 (3.4) 1477 (52.8) 1224 (43.8)
Syphilis status, *n*(%)	
Syphilis positive Syphilis negative Unknown	160 (5.7) 1322 (47.3) 1313 (47.0)
MSM status, *n* (%)	
Yes No Unknown	623 (40.6) 329 (21.4) 583 (38.0)
Study site and collection clinic type, *n* (%)	
Amedes, Germany	
• Total	529 (18.9)
• Data not available	529 (18.9)
Centrum voor Medische Analyse, Belgium	
• Total	583 (20.9)
• Primary care	583 (20.9)
Public Health Wales, UK	
• Total	986 (35.3)
• STI clinic	847 (85.9)
• Primary care	77 (7.9)
• Other	62 (6.3)
Hospital Universitario San Cecilio, Spain	
• Total	697 (24.9)
• STI clinic	427 (61.3)
• Primary care	162 (23.2)
• Hospital	62 (8.9)
• Hospital Infectious Disease Unit	46 (6.6)

*HIV*, human immunodeficiency virus; *MSM*, men who have sex with men; *SD*, standard deviation; *STI*, sexually transmitted infection

### Prevalence of MG

The prevalence of MG varied across all female sample types (*p* = 0.0042), with overall prevalence rates of 1.7% for urine samples, 2.6% for endocervical swabs, 5.7% for vaginal swabs, and 5.8% for rectal swabs (Table 2).

**Table 2:** The prevalence of *Mycoplasma* *genitalium* in female samples, by sample type and study site

Sample type	Urine		Vaginal swab					Endocervical swab				Rectal swab	
Study site	Germany (AME)	Belgium (CMA)	Germany (AME)	Wales (PHW)				Spain (USC)				Wales (PHW)	Spain (USC)
Clinic type	NA	Primary care	NA	Primary care	STI clinic	Other	Overall	Primary care	STI clinic	Other	Overall	STI clinic	STI clinic
Prevalence, % (*n*/*N*)	1.7% (5/300)	1.7% (4/238)	6.7% (1/15)	1.6% (1/62)	7.3% (15/206)	4.1% (2/49)	5.7% (18/317)	2.5% (4/161)	2.2% (2/89)	3.7% (2/54)	2.6% (8/304)	6.1% (5/82)	0.0% (0/4)
95% CI	(0.7%, 3.8%)	(0.7%, 4.2%)	(1.2%, 29.8%)	(0.3%, 8.6%)	(4.5%, 11.7%)	(1.1%, 13.7%)	(3.6%, 8.8%)	(1.0%, 6.2%)	(0.6%, 7.8%)	(1.0%, 12.5%)	(1.3%, 5.1%)	(2.6%, 13.5%)	(0.0%, 49.0%)
Overall prevalence, ^a^ % (*n*/*N*)	1.7% (9/538)		5.7% (19/332)					2.6% (8/304)				5.8% (5/86)	
95% CI	(0.9%, 3.1%)		(3.7%, 8.8%)					(1.3%, 5.15%)				(2.5%, 12.9%)	

*AME*, Amedes; *CI*, confidence interval; *CMA*, Centrum voor Medische Analyse; *NA*, not available; *PHW*, Public Health Wales, UK; *STI*, sexually transmitted infection; *USC*, Hospital Universitario San Cecilio

^a^Overall prevalence includes samples with missing data on symptom status

Overall, there was a trend to higher prevalence of MG in male samples versus female samples (*p* = 0.0024), with the overall prevalence of MG in male rectal swab samples (12.5%) higher than that in male urine samples (3.9%; Table 3, *p* < 0.0001). For the 67 MG-positive samples from PHW, 30 were from patients where both genital and rectal samples were tested. Of these 30 samples, 16 (four female, 12 male) were from eight patients where both sample types were MG-positive, 12 were from 12 male patients where only the rectal sample was positive, and two were from two female patients where only the genital sample was positive. The 37 samples where only a single sample was tested were from 17 females and 20 males. Of all the MG-positive male samples, 60 were known to be from MSM, and the characteristics of these subjects are shown in Table S1. The point prevalence rate for MG in MSM was 12.7% (46/363 [95% CI: 9.6–16.5%]) for rectal samples and 5.4% (14/260 [95% CI: 3.2– 8.8%]) for urine samples.

**Table 3:** The prevalence of *Mycoplasma genitalium* in male samples, by sample type and study site

Sample type	Urine										Rectal swab						
Study site	Germany (AME)	Belgium (CMA)	Wales (PHW)				Spain (USC)				Germany (AME)	Wales (PHW)			Spain (USC)		
Clinic type	NA	Primary care	Primary care	STI clinic	Other	Overall	Primary care	STI clinic	Other	Overall	NA	Primary care	STI clinic	Overall	STI clinic	Other	Overall
Prevalence, % (*n*/*N*)	0.5% (1/210)	5.2% (18/345)	0.0% (0/13)	3.9% (11/284)	0.0% (0/13)	3.5% (11/310)	0.0% (0/1)	5.4% (16/294)	0.0% (0/8)	5.3% (16/303)	0.0% (0/4)	0.0% (0/2)	12.0% (33/275)	11.9% (33/277)	22.5% (9/40)	8.7% (4/46)	15.1% (13/86)
95% CI	(0.1%, 2.6%)	(3.3%, 8.1%)	(0.0%, 22.8%)	(2.2%, 6.8%)	(0.0%, 22.8%)	(2.0%, 6.2%)	(0.0%, 79.3%)	(3.4%, 8.7%)	(0.0%, 32.4%)	(3.3%, 8.4%)	(0.0%, 49.0%)	(0.0%, 65.8%)	(8.7%, 16.4%)	(8.6%, 16.3%)	(12.3%, 37.5%)	(3.4%, 20.3%)	(9.1%, 24.2%)
Overall prevalence, ^a^ % (*n*/*N*)	3.9% (46/1168)										12.5% (46/367)						
95% CI	(3.0%, 5.2%)										(9.5%, 16.3%)						

*AME*, Amedes; *CI*, confidence interval; *CMA*, Centrum voor Medische Analyse; *NA*, not available; *PHW*, Public Health Wales, UK; *STI*, sexually transmitted infection; *USC*, Hospital Universitario San Cecilio

^a^Overall prevalence includes samples with missing data on symptom status

### Prevalence of TV

The prevalence of TV was low in both male and female samples across all testing sites (Tables 4 and 5), with only 12/1535 (0.8%) male and 16/1260 (1.3%) female samples testing positive. Most (11/12 male and 15/16 female) positives were detected in patients attending primary care or STI clinics. There was a slightly higher prevalence of TV in male and female rectal swab samples (1.4% and 3.5%, respectively) compared with other sample types, although these comparisons were not statistically significant (*p* = 0.1723 and *p* = 0.0901, respectively).

**Table 4:** The prevalence of *Trichomonas vaginalis* in female samples, by sample type and study site

Sample type	Urine		Vaginal swab					Endocervical swab				Rectal swab	
Study site	Germany (AME)	Belgium (CMA)	Germany (AME)	Wales (PHW)				Spain (USC)				Wales (PHW)	Spain (USC)
Clinic type	NA	Primary care	NA	Primary care	STI clinic	Other	Overall	Primary care	STI clinic	Other	Overall	STI clinic	STI clinic
Prevalence, % (*n*/*N*)	0.0% (0/300)	2.1% (5/238)	0.0% (0/15)	1.6% (1/62)	1.9% (4/206)	0.0% (0/49)	1.6% (5/317)	1.2% (2/161)	0.0% (0/89)	1.9% (1/54)	1.0% (3/304)	3.7% (3/82)	0.0% (0/4)
95% CI	(0.0%, 1.3%)	(0.9%, 4.8%)	(0.0%, 20.4%)	(0.3%, 8.6%)	(0.8%, 4.9%)	(0.0%, 7.3%)	(0.7%, 3.6%)	(0.3%, 4.4%)	(0.0%, 4.1%)	(0.3%, 9.8%)	(0.3%, 2.9%)	(1.3%, 10.2%)	(0.0%, 49.0%)
Overall prevalence, % (n/N)	0.9% (5/538)		1.5% (5/332)					1.0% (3/304)				3.5% (3/86)	
95% CI	(0.4%, 2.2%)		(0.6%, 3.5%)					(0.3%, 2.9%)				(1.2%, 9.8%)	

*AME*, Amedes; *CI*, confidence interval; *CMA*, Centrum voor Medische Analyse; *NA*, not available; *PHW*, Public Health Wales, UK; *STI*, sexually transmitted infection; *USC*, Hospital Universitario San Cecilio

**Table 5:** The prevalence of *Trichomonas vaginalis* in male samples, by sample type and study site

Sample type	Urine										Rectal swab						
Study site	Germany (AME)	Belgium (CMA)	Wales (PHW)				Spain (USC)				Germany (AME)	Wales (PHW)			Spain (USC)		
Clinic type	NA	Primary care	Primary care	STI clinic	Other	Overall	Primary care	STI clinic	Other	Overall	NA	Primary care	STI clinic	Overall	STI clinic	Other	Overall
Prevalence, % (*n*/*N*)	0.5% (1/210)	0.3% (1/345)	0.0% (0/13)	1.4% (4/284)	0.0% (0/13)	1.3% (4/310)	0.0% (0/1)	0.3% (1/294)	0.0% (0/8)	0.3% (1/303)	0.0% (0/4)	0.0% (0/2)	1.8% (5/275)	1.8% (5/277)	0.0% (0/40)	0.0% (0/46)	0.0% (0/86)
95% CI	(0.1%, 2.6%)	(0.1%, 1.6%)	(0.0%, 22.8%)	(0.5%, 3.6%)	(0.0%, 22.8%)	(0.5%, 3.3%)	(0.0%, 79.3%)	(0.1%, 1.9%)	(0.0%, 32.4%)	(0.1%, 1.8%)	(0.0%, 49.0%)	(0.0%, 65.8%)	(0.8%, 4.2%)	(0.8%, 4.2%)	(0.0%, 8.8%)	(0.0%, 7.7%)	(0.0%, 4.3%)
Overall prevalence, % (*n*/*N*)	0.6% (7/1168)										1.4% (5/367)						
95% CI	(0.3%, 1.2%)										(0.6%, 3.1%)						

*AME*, Amedes; *CI*, confidence interval; *CMA*, Centrum voor Medische Analyse; *NA*, not available; *PHW*, Public Health Wales, UK; *STI*, sexually transmitted infection; *USC*, Hospital Universitario San Cecilio

### MG and TV co-infections and prevalence across age groups

Low rates of co-infection with MG were observed in this study. In total, only eight female (0.6%) and nine male (0.6%) samples were co-infected with MG and CT, and only one female (0.1%) and six male (0.4%) samples were co-infected with MG and NG (Table S2A). HIV co-infection with MG was not reported in any female samples, while in males, three urine samples (8.8%) and 12 rectal swabs (20.7%) were also HIV positive (Table S2A), and an association between MG status and HIV status was observed for male rectal samples (*p* = 0.044). There were no reported co-infections of MG and syphilis in female samples, but seven MG-positive urine samples and 11 rectal swabs were from male samples that were co-infected with syphilis; however, the association between MG and syphilis was not significant (*p* = 0.0980; Table S2A).

Rates of TV co-infection were also very low; no female samples were co-infected with TV and HIV, syphilis, or NG, and only two female samples were co-infected with CT (Table S2B). Only one male sample was positive for both TV and HIV, two male samples were positive for TV and syphilis (both from rectal swabs; Table S2B), and no male samples were co-infected with TV and NG or CT.

**Fig. 1:**
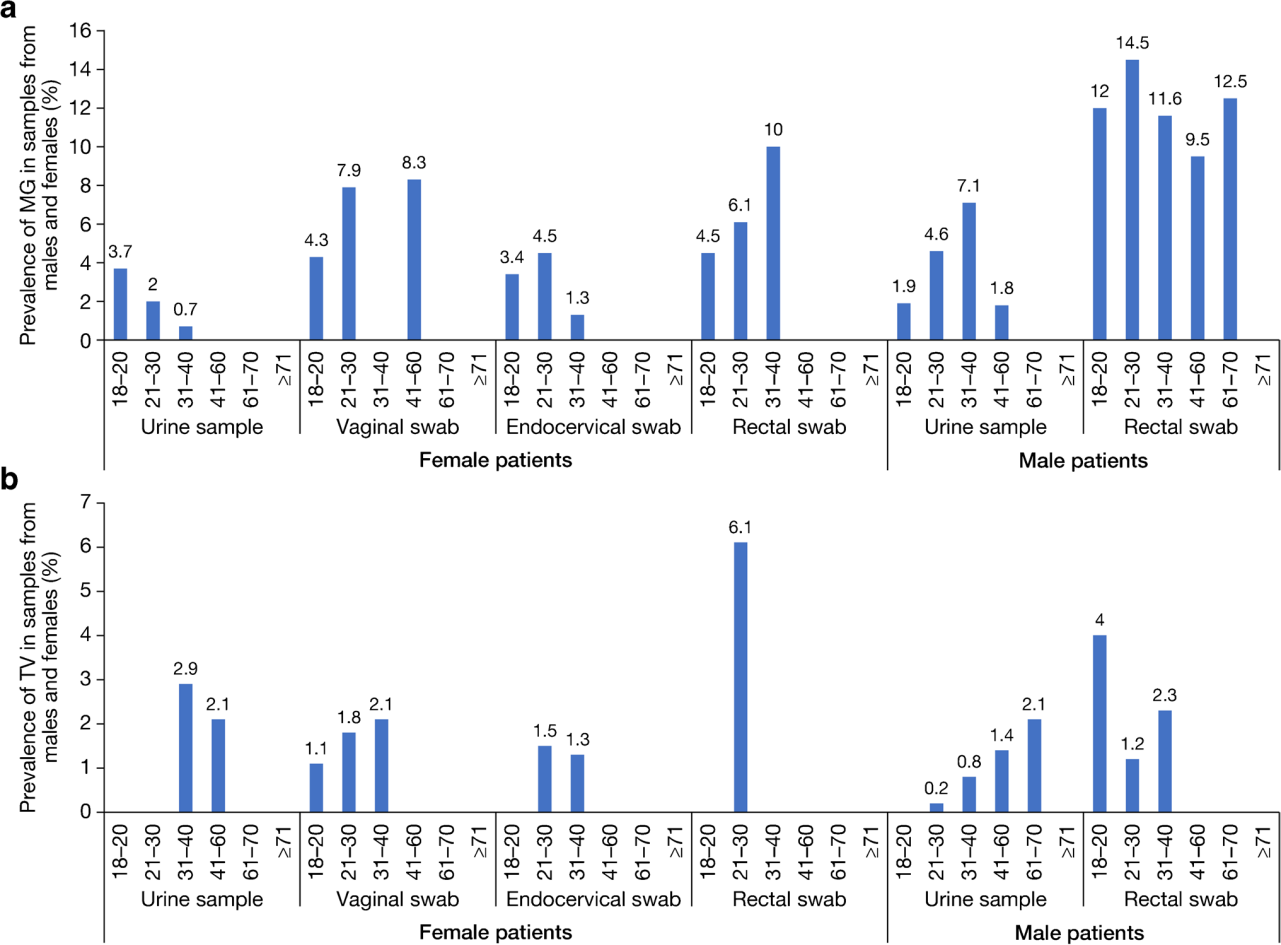
Prevalence per sample type and subject age (years) for **A** *Mycoplasma genitalium* or **B** *Trichomonas vaginalis*

While the majority of TV and MG infections were in patients aged between 21 and 40 years, positive samples were seen across most age groups (Table S3A and B; Fig. 1).

## Discussion

In this analysis of the prevalence of TV and MG, in 2795 samples indicated for STI testing at different centers throughout Europe, the point prevalence of both TV and MG was largely comparable across centers. The prevalence of MG in female samples in Wales was higher than in other collection sites; however, in Wales, the center collected only female vaginal and rectal swabs, whereas other centers collected more urine and endocervical swabs. In our study, the point prevalence rates of MG were lower in female samples than in male samples, and were similar to those reported in other European studies of patients across various settings [2, 9, 10, 13, 26]. The prevalence of TV in male and female samples in this study was comparable to published rates in France and in the USA [17, 26, 27]. TV prevalence rate was higher in females compared with males, but this difference was not significant. Many patients in the study attended STI clinics and as such are considered at high risk for MG and TV infection. While rates of TV and MG in Europe may be lower than in other regions, such as Africa [1, 6, 20, 26], higher rates of TV prevalence have been reported in other Western countries, such as the USA, compared with European countries. Estimates of TV prevalence of 1.8% and 0.5% in women and men respectively have been documented in the USA, with wide ethnic disparity (0.4–6.8%) [27]. Other studies have estimated that 3.1% of women of reproductive age are infected with TV in the USA [17], while overall TV prevalence has been estimated at 1.7% in France [26].

The results of this study have demonstrated differences in prevalence rates dependent on sample site, with rectal samples having the highest rates for MG and TV in both male and female patients, albeit this did not reach statistical significance for TV. While there are numerous studies of rectal MG prevalence, particularly in MSM [14, 28, 29], few studies have examined rectal samples for MG or TV in female patients [20, 30], and anorectal testing for STIs (CT and NG) from women is rarely done in practice [31]. In our study, prevalence of MG and TV infection in female rectal samples was higher compared with vaginal/endocervical or urine samples, which is in contrast with the results from other studies of women at high risk of STIs [20, 30]. Although, the number of female rectal samples in our study was relatively small and thus the CI is wide for the prevalence of female rectal infection. Rectal colonization by cross-reactive related species has been raised as a potential source of false-positives in NAATs for STIs, such as NG [32]; however, it is unlikely that this would explain the higher rates of MG and TV prevalence found in rectal swabs versus urine samples in this study. This is because the detection of MG using the cobas TV/MG assay does not cross-react with typical commensal bacteria and the detection of TV does not cross-react with commensals like *Trichomonas tenax* or *Pentatrichomonas homonis*, or pathogens like *Giardia lamblia* (unpublished data). However, when using simulated specimens spiked with *T. tenax* in relatively high concentrations (e.g., ≥ 1.0 × 10^6^ colony-forming unit (CFU)/mL) and *T. vaginalis* in low concentrations, it has been shown that *T. tenax* interferes with the detection of *T. vaginalis*, which could lead to false-negative results [23].

Rectal samples are useful for the detection of MG infections in MSM, since a large number of infections may be missed if this site is not sampled [2]. However, due to the high risk of antibiotic resistance in MSM, testing from rectal samples is only indicated in men with symptomatic proctitis after exclusion of NG and CT infection [2]. In our study, the point prevalence rate for MG in rectal samples from MSM was more than double that in urine samples from MSM. Similar rates of MG positivity were recorded in a Northern Irish study of 107 rectal swabs from MSM subjects negative for CT and NG, where 9.3% of samples were positive for MG [33]. The MG-positive sample data from PHW in our study indicate that MG detection (20% [12/59 patient infections) may have been missed if rectal testing was not performed. Although rectal swab testing is currently an off-label use of the cobas TV/MG assay [23], which requires validation prior to clinical application, these findings warrant further exploration of the accuracy of this assay for detection of MG in this sample type.

Positive samples for both MG and TV were reported across the majority of age groups, with most cases reported in patients aged 21–40 years in this study, which is consistent with a previous study performed in French patients [26]. The age distribution is also similar with that reported for other non-ulcerative STIs (NG and CT) in Europe [34, 35]. Increased prevalence of MG infection has been associated with patients aged under 30 years [5], while TV infections have been reported to be positively associated with increasing age [36]. Our findings show that TV infections were uncommon in patients aged over 40 years, which could be due to a non-representative study population. Indeed, prevalence of TV has been shown to vary not only with age but also in different patient groups. A UK-based study in female patients found higher prevalence of TV in STI clinics and that older age, Black ethnicity, and deprivation were independent risk factors for TV infection, indicating that different patient groups included in a study may lead to variations in prevalence [37].

Low rates of co-infection with other STIs were observed in this study for both MG and TV. Only 0.1 to 0.6% of female and male samples were co-infected with MG and CT/NG; these rates of co-infection are lower than some reports in the literature [5, 26, 38], but comparable with others within the geographical region [39]. Co-infection of MG with CT was reported at 29.9% and co-infection of MG with NG at 23.6% in young women at high risk in the USA [38], while in a study of French patients attending STI clinics, the rate of co-infection of MG with CT was 7.7% and MG with NG was 10.1% [26]. It is unclear why the co-infection rates of MG and TV with CT and NG are lower in this study than in previous studies. CT and NG infection status was not available for every specimen in the study, and it may be that CT and NG infection was underrepresented among the remnant samples used due to the need for confirmatory testing or the degradation of samples over time. Alternatively, these data may reflect the positive impact of effective screening programs for CT and NG.

Neither syphilis nor HIV and MG/TV co-infections were reported in any female patients; however, a positive, and significant, trend in HIV co-infection with MG was reported in male rectal samples (20.7%; *p* = 0.0443). In Australia, MSM living with symptomatic proctitis and HIV were more likely to have rectal MG infection (21%) than those without HIV (8%) [40], while in the USA TV infection has been observed to be less common in men living with HIV than women living with HIV and infrequently seen in HIV-infected MSM [27]. An association between MG or TV infection and HIV acquisition and transmission has been previously reported [3, 4], and ensuring adequate testing of samples to capture all cases of MG/TV and HIV co-infection is pertinent for the continued health of these patients. Several MG-positive male samples with syphilis had MG detected in urine samples and rectal swabs. Two male samples with syphilis had TV detected from rectal swabs. There are increasing rates of syphilis reported in Europe (an increase of 70% since 2010), particularly in men [41], which may explain why some male samples in our study had co-infection with syphilis and MG/TV but female samples did not.

Whilst the cobas TV/MG test is a duplex assay, it can also be run as a single analyte test depending on the clinical setting. Detecting only MG may be more useful in low TV prevalence populations. Given the rise in MG macrolide-resistant strains and the added challenge this poses for infection management and control [2, 15], it is important to further determine MG macrolide resistance-associated mutations. For this, an MG assay with multiplexing capacity may be preferable in providing MG antibiotic-resistance determinant detection in populations with low TV prevalence, such as with those in our study. Despite the cobas TV/MG assay not having the ability to detect antibiotic-resistance determinants, this assay could potentially be used for initial detection of MG, and MG-positive samples analyzed for antibiotic resistance with additional assays as required. However, such an approach would require a thorough risk-benefit and cost-effectiveness assessment to ascertain its value.

The main limitations of our study are the incomplete data and lack of definition for symptom status, and deficient patient risk factor information. Despite the inability to draw conclusions regarding symptom status in this study, for samples with available symptom data, the symptom status of MG-positive samples appeared to differ between sample sites and sex (data not shown), suggesting a possible area for future investigation.

## Conclusion

The prevalence data reported here for MG and TV infections were comparable to the published literature in Europe. Our finding that there is a higher prevalence of MG and TV in rectal swab samples compared with other sample types, particularly for male patients, suggests that there may be value in further investigation of infection rates at these sites and formally validating the use and accuracy of the cobas TV/MG assay for detecting these organisms in rectal samples.

## Supplementary Information

Below is the link to the electronic supplementary material.

Supplementary file1 (DOCX 31 KB)

## Data Availability

All data relevant to the study are included in the article or uploaded as supplementary information.
