# Supplementary material for: The prevalence of Mycoplasma genitalium (MG) and Trichomonas vaginalis (TV) at testing centers in Belgium, Germany, Spain, and the UK using the cobas TV/MG molecular assay
Source: Eur J Clin Microbiol Infect Dis. 2022 Nov 11;42(1):43–52. doi: 10.1007/s10096-022-04521-5 (PMC9816248; doi:10.1007/s10096-022-04521-5)
Supplement: Supplementary file 1 — Supplementary file1 (DOCX 31 KB) [file 10096_2022_4521_MOESM1_ESM.docx]

**Supplementary material**

**Table S1** Characteristics of *Mycoplasma genitalium*-positive samples from MSM, by sample type and study site

|  | **Urine** | | | **Rectal swab** | | | | **Overall**  **N=60** |
| --- | --- | --- | --- | --- | --- | --- | --- | --- |
|  | **PHW STI clinic**  **n=7** | **USC STI clinic**  **n=7** | **All centers**  **n=14** | **PHW STI clinic**  **n=33** | **USC STI clinic**  **n=9** | **USC hospital IDU**  **n=4** | **All centers**  **n=46** |  |
| Age, median (range) | 29 (22–48) | 32 (28–35) | 31 (22–48) | 29 (18–54) | 30 (24–53) | 41 (30–67) | 29 (18–67) | 29.5 (18–67) |
| HIV status, n (%)  Positive  Negative  Unknown/NA^a^ | 3 (42.9)  3 (42.9)  1 (14.3) | 0 (0.0)  7 (100.0)  0 (0.0) | 3 (21.4)  10 (71.4)  1 (7.1) | 9 (27.3)  17 (51.5)  7 (21.2) | 1 (11.1)  7 (77.8)  1 (11.1) | 2 (50.0)  2 (50.0)  0 (0.0) | 12 (26.1)  26 (56.5)  8 (17.4) | 15 (25.0)  36 (60.0)  9 (15.0) |
| Syphilis status, n (%)  Positive  Negative  Unknown/NA^a^ | 3 (42.9)  3 (42.9)  1 (14.3) | 2 (28.6)  5 (71.4)  0 (0.0) | 5 (35.7)  8 (57.1)  1 (7.1) | 5 (15.2)  21 (63.6)  7 (21.2) | 3 (33.3)  6 (66.7)  0 (0.0) | 3 (75.0)  1 (25.0)  0 (0.0) | 11 (23.9)  28 (60.9)  7 (15.2) | 16 (26.7)  36 (60.0)  8 (13.3) |
| CT status, n (%)  Positive  Negative  Inhibitory  Unknown/NA^a^ | 0 (0.0)  7 (100.0)  0 (0.0)  0 (0.0) | 1 (14.3)  6 (85.7)  0 (0.0)  0 (0.0) | 1 (7.1)  13 (92.9)  0 (0.0)  0 (0.0) | 0 (0.0)  32 (97.0)  1 (3.0)  0 (0.0) | 2 (22.2)  7 (77.8)  0 (0.0)  0 (0.0) | 0 (0.0)  0 (0.0)  0 (0.0)  4 (100.0) | 2 (4.3)  39 (84.8)  1 (2.2)  4 (8.7) | 3 (5.0)  52 (86.7)  1 (1.7)  4 (6.7) |
| NG status, n (%)  Positive  Negative  Unknown/NA^a^ | 0 (0.0)  7 (100.0)  0 (0.0) | 1 (14.3)  6 (85.7)  0 (0.0) | 1 (7.1)  13 (92.9)  0 (0.0) | 2 (6.1)  31 (93.9)  0 (0.0) | 3 (33.3)  6 (66.7)  0 (0.0) | 0 (0.0)  0 (0.0)  4 (100.0) | 5 (10.9)  37 (80.4)  4 (8.7) | 6 (10.0)  50 (83.3)  4 (6.7) |
| TV status, n (%)  Positive  Negative  Unknown/NA^a^ | 0 (0.0)  7 (100.0)  0 (0.0) | 0 (0.0)  7 (100.0)  0 (0.0) | 0 (0.0)  14 (100.0)  0 (0.0) | 1 (3.0)  32 (97.0)  0 (0.0) | 0 (0.0)  9 (100.0)  0 (0.0) | 0 (0.0)  4 (100.0)  0 (0.0) | 1 (2.2)  45 (97.8)  0 (0.0) | 1 (1.7)  59 (98.3)  0 (0.0) |

AME, Amedes, Germany; CI, confidence interval; CMA, Centrum voor Medische Analyse, Belgium; CT, *Chlamydia trachomatis*; HIV, human immunodeficiency virus; IDU, infectious disease unit; MSM, men who have sex with men; NA, not available; NG, *Neisseria gonorrhoeae*; PHW, Public Health Wales, UK; STI, sexually transmitted infection; TV, *Trichomonas vaginalis*; USC, Hospital Universitario San Cecilio, Spain.

^a^Unknown/NA category indicates subjects who reported "unknown" or had missing data.

**Table S2A** The prevalence of *Mycoplasma genitalium* in female and male samples, and the prevalence of other infections

|  | **Female samples** | | | | **Male samples** | |
| --- | --- | --- | --- | --- | --- | --- |
| Sample type | Urine | Vaginal swab | Endocervical swab | Rectal swab | Urine | Rectal swab |
| Overall prevalence, ^a^ % (n/N) | 1.7% (9/538) | 5.7% (19/332) | 2.6% (8/304) | 5.8% (5/86) | 3.9% (46/1168) | 12.5% (46/367) |
| 95% CI | (0.9%, 3.1%) | (3.7%, 8.8%) | (1.3%, 5.15%) | (2.5%, 12.9%) | (3.0%, 5.2%) | (9.5%, 16.3%) |
| Prevalence HIV positive, % (n/N) | NA | 0.0% (0/2) | NA | NA | 8.8% (3/34) | 20.7% (12/58) |
| 95% CI | NA | (0.0%, 65.8%) | NA | NA | (3.0%, 23.0%) | (12.3%, 32.8%) |
| Prevalence CT positive, % (n/N) | 4.5% (2/44) | 5.3% (1/19) | 19.2% (5/26) | 0.0% (0/4) | 9.3% (7/75) | 9.1% (2/22) |
| 95% CI | (1.3%, 15.1%) | (0.9%, 24.6%) | (8.5%, 37.9%) | (0.0%, 49.0%) | (4.6%, 18.0%) | (2.5%, 27.8%) |
| Prevalence NG positive, % (n/N) | 0.0% (0/1) | 0.0% (0/6) | 100.0% (1/1) | 0.0% (0/1) | 4.5% (1/22) | 11.9% (5/42) |
| 95% CI | (0.0%, 79.3%) | (0.0%, 39.0%) | (20.7%, 100.0%) | (0.0%, 79.3%) | (0.8%, 21.8%) | (5.2%, 25.0%) |
| Prevalence syphilis positive, % (n/N) | NA | NA | 0.0% (0/4) | NA | 8.3% (7/84) | 15.3% (11/72) |
| 95% CI | NA | NA | (0.0%, 49.0%) | NA | (4.1%, 16.2%) | (8.8%, 25.3%) |

CI, confidence interval; CT, *Chlamydia trachomatis*; HIV, human immunodeficiency virus; NA, not available; NG, *Neisseria gonorrhoeae*.

^a^Overall prevalence includes samples with missing data on symptom status.

**Table S2B** The prevalence of *Trichomonas vaginalis* in female and male samples, and the prevalence of other infections

|  | **Female samples** | | | | **Male samples** | |
| --- | --- | --- | --- | --- | --- | --- |
| Sample type | Urine | Vaginal swab | Endocervical swab | Rectal swab | Urine | Rectal swab |
| Overall prevalence, % (n/N) | 0.9% (5/538) | 1.5% (5/332) | 1.0% (3/304) | 3.5% (3/86) | 0.6% (7/1168) | 1.4% (5/367) |
| 95% CI | (0.4%, 2.2%) | (0.6%, 3.5%) | (0.3%, 2.9%) | (1.2%, 9.8%) | (0.3%, 1.2%) | (0.6%, 3.1%) |
| Prevalence HIV positive, % (n/N) | NA | 0.0% (0/2) | NA | NA | 0.0% (0/34) | 1.7% (1/58) |
| 95% CI | NA | (0.0%, 65.8%) | NA | NA | (0.0%, 10.2%) | (0.3%, 9.1%) |
| Prevalence CT positive, % (n/N) | 0.0% (0/44) | 5.3% (1/19) | 3.8% (1/26) | 0.0% (0/4) | 0.0% (0/75) | 0.0% (0/22) |
| 95% CI | (0.0%, 8.0%) | (0.9%, 24.6%) | (0.7%, 18.9%) | (0.0%, 49.0%) | (0.0%, 4.9%) | (0.0%, 14.9%) |
| Prevalence NG positive, % (n/N) | 0.0% (0/1) | 0.0% (0/6) | 0.0% (0/1) | 0.0% (0/1) | 0.0% (0/22) | 0.0% (0/42) |
| 95% CI | (0.0%, 79.3%) | (0.0%, 39.0%) | (0.0%, 79.3%) | (0.0%, 79.3%) | (0.0%, 14.9%) | (0.0%, 8.4%) |
| Prevalence syphilis positive, % (n/N) | NA | NA | 0.0% (0/4) | NA | 0.0% (0/84) | 2.8% (2/72) |
| 95% CI | NA | NA | (0.0%. 49.0%) | NA | (0.0%, 4.4%) | (0.8%, 9.6%) |

CI, confidence interval; CT, *Chlamydia trachomatis*; HIV, human immunodeficiency virus; NA, not available; NG, *Neisseria gonorrhoeae*; STI, sexually transmitted infection.

**Table S3A** *Mycoplasma genitalium* prevalence per sample type and subject age

| Samples | Age category (years) | Prevalence, % (n/N) | 95% CI (%) |
| --- | --- | --- | --- |
| Female |  |  |  |
| Urine sample | 18–20 | 3.2 (3/93) | 1.1–9.1 |
|  | 21–30 | 2.0 (5/253) | 0.8–4.5 |
|  | 31–40 | 0.7 (1/138) | 0.1–4.0 |
|  | 41–60 | 0.0 (0/47) | 0.0–7.6 |
|  | 61–70 | 0.0 (0/7) | 0.0–35.4 |
|  | ≥71 | - | - |
| Vaginal swab | 18–20 | 4.3 (4/94) | 1.7–10.4 |
|  | 21–30 | 7.9 (13/165) | 4.7–13.0 |
|  | 31–40 | 0.0 (0/47) | 0.0–7.6 |
|  | 41–60 | 8.3 (2/24) | 2.3–25.8 |
|  | 61–70 | 0.0 (0/1) | 0.0–79.3 |
|  | ≥71 | 0.0 (0/1) | 0.0–79.3 |
| Endocervical swab | 18–20 | 3.4 (1/29) | 0.6–17.2 |
|  | 21–30 | 4.5 (6/134) | 2.1–9.4 |
|  | 31–40 | 1.3 (1/76) | 0.2–7.1 |
|  | 41–60 | 0.0 (0/56) | 0.0–6.4 |
|  | 61–70 | 0.0 (0/4) | 0.0–49.0 |
|  | ≥71 | 0.0 (0/5) | 0.0–43.4 |
| Rectal swab | 18–20 | 4.5 (1/22) | 0.8–21.8 |
|  | 21–30 | 6.1 (3/49) | 2.1–16.5 |
|  | 31–40 | 10.0 (1/10) | 1.8–40.4 |
|  | 41–60 | 0.0 (0/5) | 0.0–43.4 |
|  | 61–70 | - | - |
|  | ≥71 | - | - |
| Male |  |  |  |
| Urine sample | 18–20 | 1.9 (2/105) | 0.5–6.7 |
|  | 21–30 | 4.6 (22/483) | 3.0–6.8 |
|  | 31–40 | 7.1 (18/252) | 4.6–11.0 |
|  | 41–60 | 1.8 (4/220) | 0.7–4.6 |
|  | 61–70 | 0.0 (0/48) | 0.0–7.4 |
|  | ≥71 | 0.0 (0/60) | 0.0–6.0 |
| Rectal swab | 18–20 | 12.0 (3/25) | 4.2–30.0 |
|  | 21–30 | 14.5 (25/172) | 10.0–20.6 |
|  | 31–40 | 11.6 (10/86) | 6.4–20.1 |
|  | 41–60 | 9.5 (7/74) | 4.7–18.3 |
|  | 61–70 | 12.5 (1/8) | 2.2–47.1 |
|  | ≥71 | 0.0 (0/2) | 0.0–65.8 |

**Table S3B** *Trichomonas vaginalis* prevalence per sample type and subject age

| Samples | Age category (years) | Prevalence, % (n/N) | 95% CI (%) |
| --- | --- | --- | --- |
| Female |  |  |  |
| Urine sample | 18–20 | 0.0 (0/93) | 0.0–4.0 |
|  | 21–30 | 0.0 (0/253) | 0.0–3.5 |
|  | 31–40 | 2.9 (4/138) | 1.1–7.2 |
|  | 41–60 | 2.1 (1/47) | 0.4–11.1 |
|  | 61–70 | 0.0 (0/7) | 0.0–35.4 |
|  | ≥71 | - | - |
| Vaginal swab | 18–20 | 1.1 (1/94) | 0.2–5.8 |
|  | 21–30 | 1.8 (3/165) | 0.6–5.2 |
|  | 31–40 | 2.1 (1/47) | 0.4–11.1 |
|  | 41–60 | 0.0 (0/24) | 0.0–13.8 |
|  | 61–70 | 0.0 (0/1) | 0.0–79.3 |
|  | ≥71 | 0.0 (0/1) | 0.0–79.3 |
| Endocervical swab | 18–20 | 0.0 (0/29) | 0.0–11.7 |
|  | 21–30 | 1.5 (2/134) | 0.4–5.3 |
|  | 31–40 | 1.3 (1/76) | 0.2–7.1 |
|  | 41–60 | 0.0 (0/56) | 0.0–6.4 |
|  | 61–70 | 0.0 (0/4) | 0.0–49.0 |
|  | ≥71 | 0.0 (0/5) | 0.0–43.4 |
| Rectal swab | 18–20 | 0.0 (0/22) | 0.0–14.9 |
|  | 21–30 | 6.1 (3/49) | 2.1–16.5 |
|  | 31–40 | 0.0 (0/10) | 0.0–27.8 |
|  | 41–60 | 0.0 (0/5) | 0.0–43.4 |
|  | 61–70 | - | - |
|  | ≥71 | - | - |
| Male |  |  |  |
| Urine sample | 18–20 | 0.0 (0/105) | 0.0–3.5 |
|  | 21–30 | 0.2 (1/483) | 0.0–1.2 |
|  | 31–40 | 0.8 (2/252) | 0.2–2.8 |
|  | 41–60 | 1.4 (3/220) | 0.5–3.9 |
|  | 61–70 | 2.1 (1/48) | 0.4–10.9 |
|  | ≥71 | 0.0 (0/60) | 0.0–6.0 |
| Rectal swab | 18–20 | 4.0 (1/25) | 0.7–19.5 |
|  | 21–30 | 1.2 (2/172) | 0.3–4.1 |
|  | 31–40 | 2.3 (2/86) | 0.6–8.1 |
|  | 41–60 | 0.0 (0/74) | 0.0–4.9 |
|  | 61–70 | 0.0 (0/8) | 0.0–32.4 |
|  | ≥71 | 0.0 (0/2) | 0.0–65.8 |

CI, confidence interval
